# Osteoma Cutis and Tonsillolith: A Cone Beam Computed Tomography Study

**DOI:** 10.7759/cureus.3003

**Published:** 2018-07-19

**Authors:** Ahmed Z Abdelkarim, Scott Lozanoff, Shaimaa M Abu el Sadat, Ali Z Syed

**Affiliations:** 1 Department of Anatomy, Biochemistry & Physiology, University of Hawaii School of Medicine, Honolulu, USA; 2 Department of Oral Radiology, Ain-Shams University, Cairo, EGY; 3 Department of Oral Medicine and Diagnostic Sciences, CWRU School of Dental Medicine, Cleveland, USA

**Keywords:** osteoma cutis, diagnostic imaging, tonsilloliths., cone beam

## Abstract

In this study, we report a rare case of osteoma cutis (OC) and tonsillolith, diagnosed using cone beam computed tomography. The dystrophic calcifications in the face and tonsils were incidentally found during examination of the patient’s scan with no relation to the main chief complaint. The diagnosis was OC, combined with dystrophic calcification of the tonsils. It is important to mention that OC is a rare soft-tissue ossification of cutaneous tissue, typically on the face and clinically asymptomatic. It may be primary but the majority of cases are secondary. Incidental finding of OC and tonsilloliths on a two-dimensional dental radiograph does not provide sufficient information concerning the location of these calcifications. Thus, cone beam computed tomography (CBCT) provides critical information for the diagnosis of asymptomatic OC lesions not available through any other means of clinical detection.

## Introduction

Osteoma cutis (OC) and tonsilloliths are soft-tissue calcifications in the head and neck region that result from the deposition of minerals at specific sites and may be of pathological, age-related or idiopathic origins [1]. OC is a rare, benign ectopic presence of calcification within the dermis or epidermis with non-invasive behavior [2]. It is indicative of Albright syndrome, where mesenchymal cells secrete a matrix that becomes calcified and differentiates into focal bone in the soft tissues. The most common form, about 85% of the detected cases, is secondary to long duration conditions such as acne, chronic inflammatory dermatoses, or scarring [1]. Previous studies reported that OC manifests a broad spectrum of features; [2-5] it has been described clinically as a normochrome skin over subcutaneous non-compressible papules with a higher incidence in females and/or in patients that have been previously diagnosed with acne vulgaris [6].

Another example of soft-tissue calcification is tonsillar stones or tonsilloliths. These lesions occur as white or yellow densifications in tonsillar crypts that originate from microorganism and tissue debris retention in the crypts of palatine tonsils [7]. Tonsilloliths arise as calcifications resulting from repeated inflammation of the tonsillar crypts during recurrent tonsillitis without gender prevalence [8].

In dentistry, routinely performed dental radiographs can capture the lesion most commonly in the lips and cheek regions; however, due to difficulty in localization, interpretation of OC cases has always been challenging especially using conventional imaging. CBCT, with its 3D software based volumetric reconstruction and multiplanar evaluation with a distortion-free image, offers an important clinical tool facilitating detection and diagnosis of these calcific lesions [9]. Cone beam computed tomography (CBCT) is particularly useful since a typical craniofacial scan depicts areas beyond one specific region of interest thus facilitating and enabling incidental findings. For example, Safi et al. recently reported the prevalence of OC as an incidental finding detected on CBCT to be 2.27% [10].

The present case reports OC in a combination of tonsillar stones. This condition was an incidental finding identified during a CBCT scan evaluation, apart from the main chief complaint of the patient without related symptoms. The purpose of this presentation is to underscore the importance of CBCT for evaluating OC lesions by the dental professional using three-dimensional radiography.

## Case presentation

A 33-year-old male presented to be admitted as a patient at Case Western Reserve University School of Dental Medicine clinic in Cleveland, Ohio for the purpose of obtaining dental implants. However, the patient complained of pain in his upper jaw bilaterally. His medical history was free from systemic diseases but indicated a past history of acne and recurrent tonsillitis. His vital signs were recorded as 126/85 mmHg blood pressure, pulse of 103 bpm, 15 respirations per minute, height 6.1 ft, weight 196 lb, a calculated body mass index (BMI) of 23.71. No other medical conditions were listed, and the patient did not report taking any medications.

**Figure 1:**
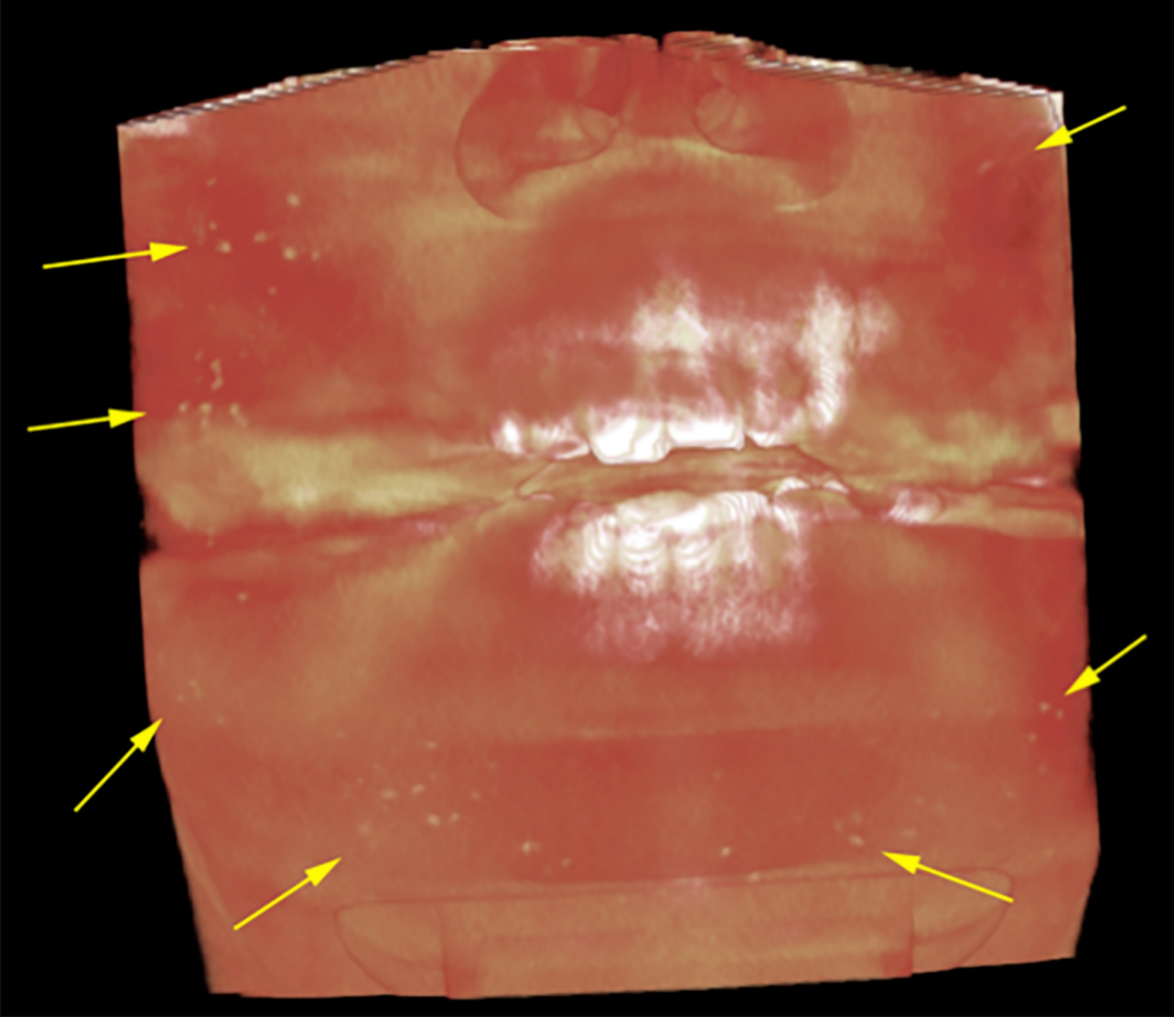
Volumetric rendering of CBCT scan of OC. Soft tissue rendering shows multiple small nodules of high density spread in the cheeks, lips and chin regions indicated by yellow arrows.

Upon intraoral examination, badly decayed upper right second molar and highly restored left second molar were noticed with pain on percussion. Dental periapical lesions were suspected. The patient was referred to a private dental imaging center for imaging of the jaws for implant treatment planning. A CBCT scan was taken using a Planmeca Promax X-ray (Planmeca, Helsinki, Finland) unit. Upon reviewing the scan, multiple small nodules of high density were identified as an incidental finding during the interpretation of the patient’s scan (Figure 1).

**Figure 2:**
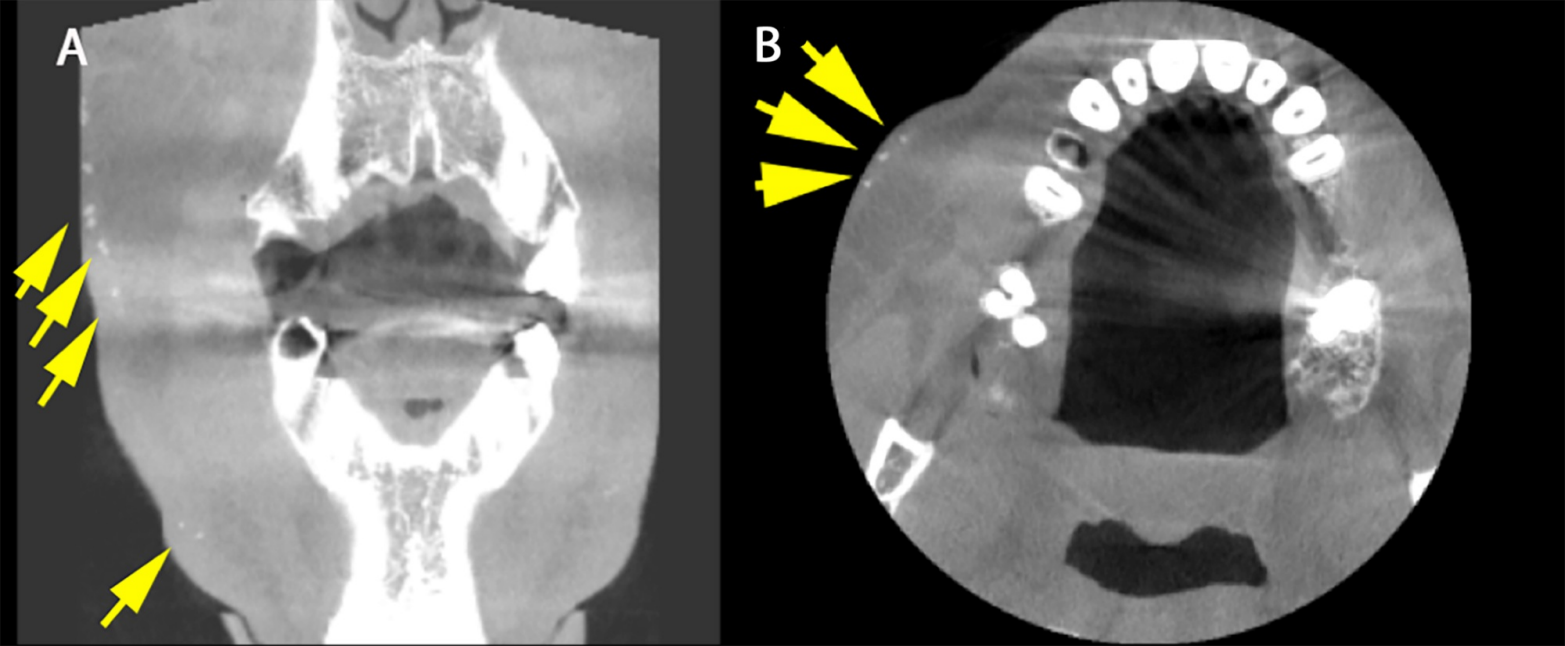
Axial and coronal sections. A) Coronal cut showing multiple radiopaque masses along the dermal layer lateral to the buccinator muscle indicated by yellow arrows. B) Axial cut with a yellow arrows showing 3 small concentric nodules in the buccal cheek

The nodules appear to be dispersed among the layers of the face, which were clearly displayed in more than coronal and axial cuts (Figure 2).

**Figure 3:**
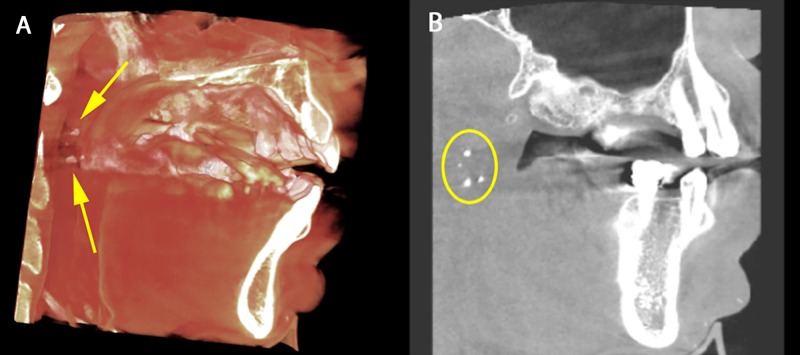
Tonsillar stones in the palatine tonsils. A) Volumetric rendering displaying multiple small radiopaque masses in the left palatine tonsils consistent with tonsilloliths (yellow arrows). B) Sagittal section displaying multiple small radiopaque masses in the left palatine tonsils consistent with tonsilloliths (yellow circle).

The multiple nodules were consistent with the miliary type of OC and his past history of acne as a teenager. The palatine tonsils, in addition, revealed radiopaque masses bilaterally, but more numerous and prominent on the left side. The calcifications were also consistent with the patient history of recurrent tonsillitis as tonsillar stones (tonsilloliths) (Figure 3). The patient was advised to visit his dentist and receive a panoramic radiography regularly to follow up the spread of the calcification, especially to the blood vessels.

## Discussion

The case reported in this study is that of OC with tonsilloliths, diagnosed using CBCT. OC is a benign and rare condition in which soft-tissue ossifications occur in the dermis layer of skin. The disease is categorized into primary or secondary forms. Primary forms are characterized by the lack of any history that would lead to predispose bone deposition within the layers of the skin, such as trauma or cutaneous disease, and accounts for 15% of OC cases [11]. Secondary OC, on the other hand, is associated with a known predisposing factor such as inflammation, trauma, neoplastic changes, nevi, or venous stasis [12-13].

Theories, ranging from hamartomas to nevoid tumors, have been associated with the etiology and pathogenesis of OC [14]. Also, it has been advocated that OC is the result of osteoblastic metaplasia of mesenchymal cells following prolonged inflammation associated with common acne or Botox injecting sites in the face but the specific mechanism remains unknown and debated [14].

Clinically, OC presents with different asymptomatic forms. It may present as single or multiple papules, nodules, or plaques or as miliary lesions [9]. The lesions are hard bony structures on palpation and show discoloration of the skin in occasional cases [6]. In radiographs, the lesions have a small, smoothly outlined hyperdense spots which are donut- or snowflake-like in shape and vary in size from 0.1 cm to 5.0 cm [9].

Understanding the radiographic presentation of OC facilitates definite identification since several imaging features may be mimicked by other soft-tissue calcification conditions confounding accurate diagnosis. For example, myositis ossificans, calcinosis cutis, osteoma mucosae, and dermal fillers may have the same radiographic manifestations [9, 15-16]. Other condition such as calcified phleboliths in hemangiomas, surgical clips, wires, or sutures placed for procedures such as face-lifts may be confused with OC [17-18]. Medical history and imaging findings typically provide complementary information facilitating an accurate diagnosis [19].

In this case report, OC is combined with tonsilloliths. The specific etiology and pathogenesis of tonsilloliths remain unknown, however, it is generally agreed that unresolved tonsillitis is the main cause [8]. Recurrent episodes of inflammation may cause fibrosis at the openings of the tonsillar crypts, causing microorganism and epithelial debris accumulation that leads to retention cysts formation then subsequent calcification [20]. This explanation is consistent with a patient history of recurrent tonsillitis.

Differential diagnosis of tonsilloliths includes mostly sialoliths, lymph node calcification, and phleboliths. CBCT images provide bilateral views of the jaw and are useful to determine and differentiate the locations of these calcifications [9].

Correct diagnosis of the different types of soft-tissue calcifications requires sufficient knowledge from the dental professional. Clinical management of these conditions must be based on the patient’s medical history and symptomatology [19]. Volumetric imaging has revolutionized the dental practice by making it possible to visualize structures in all three dimensions. With this privilege comes the responsibility of recognizing every small detail captured within these images. It is vital to examine the entire CBCT volume for any abnormalities and to recognize and report incidental findings. This will lead to more accurate diagnosis, appropriate management, and prompt referral of patients and improved dental health [20].

## Conclusions

In this case report, we presented a rare case of OC, combined with tonsillolith using CBCT as a diagnostic method. The general dentist, using three-dimensional dental radiography, typically encounters soft-tissue calcifications. Based on careful examination of the patient’s scan, incidental findings are adding to the value of using CBCT as a diagnostic imaging tool and ultimately, improved patient health.
